# Primary care experiences of family caregivers using the same medical facility as their care recipient or a different facility

**DOI:** 10.1002/jgf2.70000

**Published:** 2025-04-18

**Authors:** Gen Nakayama, Shoichi Masumoto, Junji Haruta, Tetsuhiro Maeno

**Affiliations:** ^1^ Department of Primary Care and Medical Education, Institute of Medicine University of Tsukuba Tsukuba Japan; ^2^ Department of Family Medicine, General Practice and Community Health, Institute of Medicine University of Tsukuba Tsukuba Japan; ^3^ Department of General Medicine Tsukuba Central Hospital Ushiku Japan; ^4^ Medical Education Center, School of Medicine Keio University Tokyo Japan; ^5^ Center for General Medicine Education, School of Medicine Keio University Tokyo Japan

**Keywords:** caregivers, healthcare quality assurance, patient experience, person‐centered care, primary health care, usual source of care

## Abstract

**Background:**

Family caregivers and care recipients do not always have the same usual source of care, which may create barriers to providing optimal care for caregivers. This study aimed to compare family caregivers' primary care experiences based on whether they used the same medical facility as their care recipient or a different facility.

**Methods:**

We used cross‐sectional data from a survey conducted in Japan in 2020. Participants were family caregivers aged 40–74 years who cared for community‐dwelling adults with chronic conditions. Caregivers' primary care experiences were assessed using the Japanese version of the Primary Care Assessment Tool Short Form (JPCAT‐SF). Multivariable linear regression analyses were conducted to evaluate differences in JPCAT‐SF scores between caregivers who used the same medical facility as their care recipient and those who used a different facility.

**Results:**

Of the 406 family caregivers analyzed, 216 (53.2%) used a different medical facility from their care recipient. After adjusting for possible confounders, JPCAT‐SF total scores were significantly lower among family caregivers using a different facility compared with those using the same facility (adjusted mean difference −5.73, 95% confidence interval: −8.93 to −2.54). The JPCAT‐SF subscale scores for longitudinality, comprehensiveness (services available), and community orientation were significantly lower in the different facility group than the same facility group.

**Conclusions:**

Family caregivers who used a different medical facility from their care recipient reported more negative primary care experiences than caregivers using the same facility. Greater efforts may be needed to provide patient‐centered, family‐oriented primary care for these caregivers.

## Graphical abstract

Our study found that family caregivers who used a different medical facility from their care recipient reported more negative primary care experiences than caregivers using the same facility. Greater efforts may be needed to provide patient‐centered, family‐oriented primary care for these caregivers.

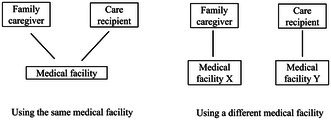

## 1 INTRODUCTION

Global population aging and the rising prevalence of chronic illnesses mean family caregivers play an increasingly vital role in supporting older adults in their daily activities.^1^ Family caregivers, often referred to as informal carers, are typically unpaid family members or close individuals who provide practical assistance to an unwell or older relative unable to perform daily activities. Compared with noncaregivers, family caregivers face heightened physical and psychological health challenges.^2^ Therefore, their well‐being is an important consideration for healthcare providers and it is crucial for healthcare systems to support family caregivers, particularly in primary care settings where interactions are frequent.^3^, ^4^

Patients' experiences in primary care offer an indicator of person‐centeredness.^5^ For family caregivers, this refers to their experiences when receiving primary care as patients. Family caregivers have reported worse primary care experiences compared with noncaregivers.^6^ In addition, it has been suggested that good primary care experiences among family caregivers may lower their caregiving stress levels.^7^ Therefore, enhancing family caregivers' primary care experiences is central to providing appropriate support for this group.

**FIGURE 1:**
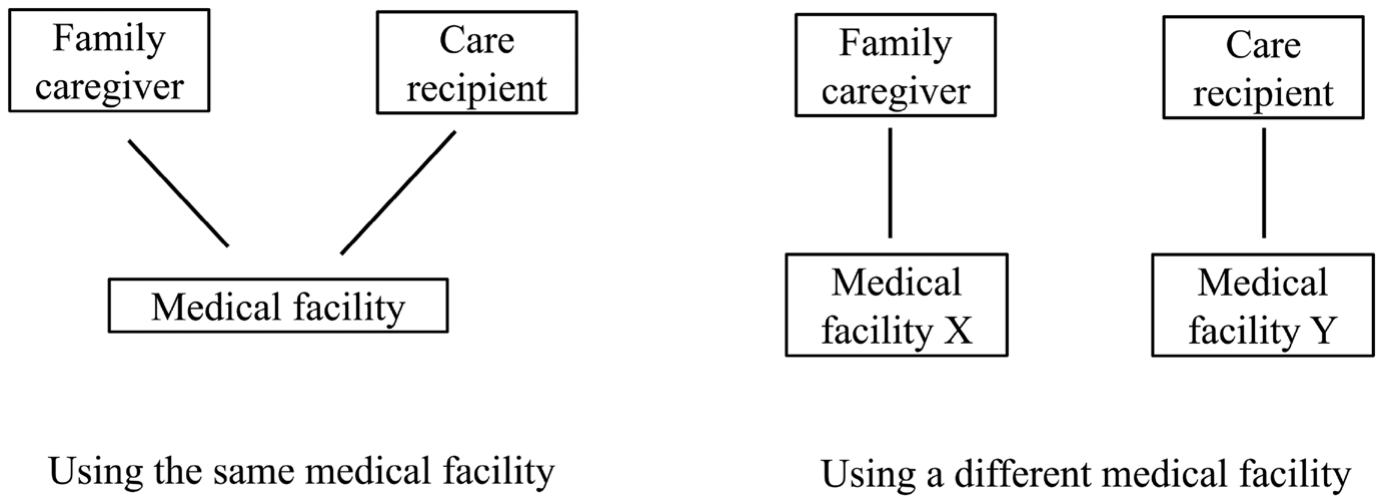
Caregivers and care recipients using the same medical facility or a different facility.

Identifying patients with caring responsibilities is a key factor that enables primary care providers to provide adequate care for family caregivers.^3^, ^8^ However, barriers to identifying such patients (i.e., family caregivers) include several factors, one of which is that they do not always have the same usual source of care (USC) as their care recipient.^9^ Figure 1 demonstrates how it can be more challenging for healthcare providers to recognize that a family caregiver is responsible for someone's care when they use a different medical facility from their care recipient. A UK study reported that less than half of the general practitioners (GPs) surveyed felt confident in identifying caregivers.^10^ That study highlighted that in addition to complicating caregiver identification, the lack of a shared GP or primary care facility between caregivers and their care recipients hindered communication between GPs and caregivers regarding the social issues related to caregiving responsibilities.^10^ In Japan, people can visit any primary care facility without restrictions or additional out‐of‐pocket costs; therefore, the lack of shared medical facility use between caregivers and their care recipients may be more prevalent than in other countries.

Despite the potential impact on family caregivers' primary care experiences when they use a different medical facility from their care recipient, to our knowledge, no studies have directly investigated this issue. To address this gap, our study aimed to compare the primary care experiences of family caregivers based on whether they used the same medical facility as their care recipient or a different facility. By examining any differences in experience, we hoped to provide insights to inform efforts to improve the care provided for family caregivers in primary care settings.

## 2 METHODS

### 2.1 Design, setting, participants, and procedures

We used cross‐sectional data from a survey conducted in Ibaraki Prefecture, which is located in the northeastern part of the Greater Tokyo Area. The survey recruited family caregivers who provided care for community‐dwelling adults with chronic illnesses. These care recipients were using Japan's Long‐Term Care Insurance (LTCI) system. Family caregivers were recruited through care managers, who are professionals that assist both caregivers and care recipients under the LTCI system. Data were collected during November and December 2020 using a self‐administered questionnaire. Family caregivers provided informed consent to participate via the questionnaire and mailed completed questionnaires to our university.

#### 2.1.1 Inclusion criteria

Participants were eligible for the survey if they were aged 40–74 years and had been caring for a care recipient using LTCI services for at least 1 year. The rationale underlying these criteria was explained in detail in a previous paper.^11^

#### 2.1.2 Exclusion criteria

Family caregivers that cared for multiple people were instructed to focus on the most dependent person; therefore, they were excluded if they completed more than one questionnaire. Family caregivers that provided care “once or less in several days”^12^ were also excluded, because those who provided less frequent care were considered to be heterogeneous. These exclusion criteria were aligned with those used in previous studies that drew on data from the same survey.^11^, ^13^ In addition, family caregivers without a USC were excluded, as this information was required in the scale used to assess their primary care experiences (see below).

### 2.2 Measures

#### 2.2.1 Family caregivers' primary care experiences

We measured family caregivers' primary care experiences using the Japanese version of the Primary Care Assessment Tool Short Form (JPCAT‐SF).^14^ The original JPCAT is a widely recognized patient‐reported experience measure in primary care, and was developed based on the Primary Care Assessment Tool from the Johns Hopkins Primary Care Policy Center.^15^, ^16^ The JPCAT‐SF simplified the response process by including fewer items than the original scale. It starts by determining whether the individual has a USC. If the respondent answers affirmatively, the tool assesses their primary care experience using 13 items. The scoring range is 0–100 points.

The 13‐item JPCAT‐SF comprises six multi‐item subscales, each representing key attributes of primary care: first contact, longitudinality, coordination, comprehensiveness (services available), comprehensiveness (services provided), and community orientation. A brief explanation for each subscale is as follows.

- *First contact* is closely related to “access to care” and measured by two items related to out‐of‐hours care.
- *Longitudinality* is assessed by two items that ask whether the respondent had experienced their physician knowing them as a whole person and knowing what their most important problems were.
- *Coordination* is evaluated by three items asking about the respondent's experiences of referral to a specialist. If the respondent had never been referred or was unsure, they received a default score of 50 points (scale midpoint).
- *Comprehensiveness (services available)* is assessed by two items that ask whether counseling for abuse and end‐of‐life preferences were available at the physician's office.
- *Comprehensiveness (services provided)* is evaluated by two items that ask whether advice on over‐the‐counter medications and medical information from media sources were discussed during visits to the physician.
- Community orientation is assessed by two items that gauge whether the physician is concerned about the needs and health issues of the community.

The JPCAT‐SF total score is the average of the six subscale scores and reflects the respondent's overall primary care experience. Higher scores indicate a better experience (range: 0–100). A previous study showed that the JPCAT‐SF had good reliability and validity.^14^

#### 2.2.2 Use of the same or a different medical facility

We asked participants about the relationship between their USC and that of their care recipient. The response options were: (1) same physician, (2) same medical facility but different physician, and (3) different medical facility. The first two options were categorized as “same medical facility.”

#### 2.2.3 Covariates

Covariates were selected for their known associations with patient preferences for medical facility choice and with patient experience. We included age, gender, educational attainment, annual household income, and self‐rated health as covariates. Among these covariates, only age was collected as an open‐ended response.

### 2.3 Statistical analyses

We calculated descriptive statistics for participants' characteristics and JPCAT‐SF scores. Bivariable and multivariable linear regression analyses were used to examine the association between using the same or a different medical facility and family caregivers' primary care experiences. The multivariable model included several confounders: age, gender, educational attainment, annual household income, and self‐rated health. By categorizing age, which was a continuous variable, all covariates were treated as categorical variables.

In addition, we investigated the association between using the same or a different medical facility and the scores for each of the JPCAT‐SF subscales. Comparisons were repeated without Bonferroni correction.^17^ Based on previous studies that used the JPCAT and JPCAT‐SF, a difference exceeding 3 points was considered a clinically meaningful difference.^18^, ^19^, ^20^, ^21^ We accounted for missing data in independent and dependent variables using multiple imputations, with 20 imputations by fully conditional specification. All statistical analyses were conducted using SPSS Statistics version 29 (IBM Corp, Armonk, NY, USA).

## 3 RESULTS

### 3.1 Participants' characteristics

**FIGURE 2:**
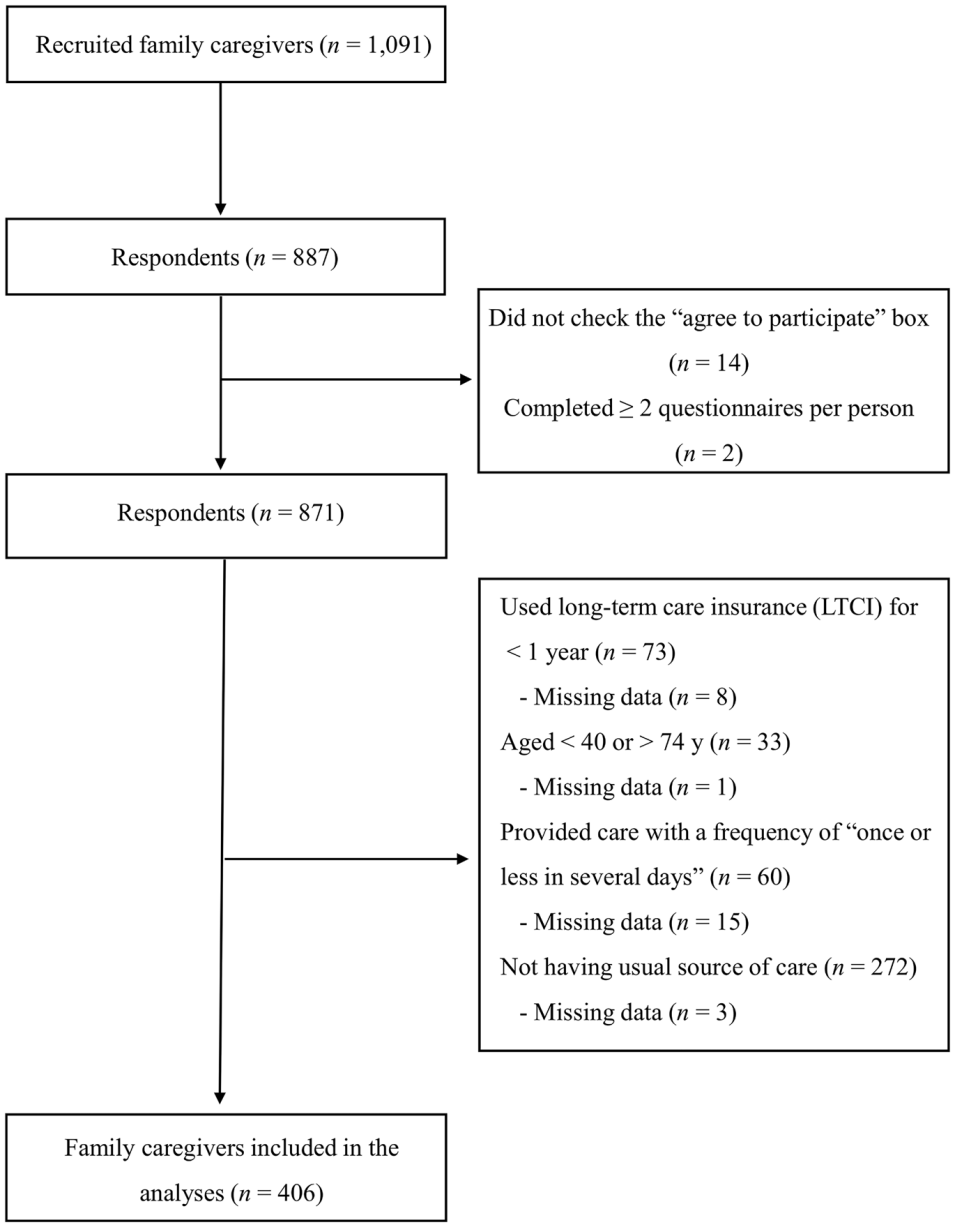
Flow chart of participants.

Of the 1091 family caregivers recruited for the survey, 887 (81.3%) responded to the questionnaire, and 406 caregivers were ultimately included in our analyses. Figure 2 shows the flow of study participants. Table 1 summarizes participants' characteristics by whether they used the same medical facility as their care recipients or a different facility. Of the 406 caregivers analyzed, 186 (45.8%) used the same medical facility as their care recipient, with 149 having the same physician and 37 seeing a different physician at the same facility. Family caregivers who used a different medical facility from their care recipient tended to have lower scores for the first contact, longitudinality, comprehensiveness (services available), and community orientation subscales than those who used the same facility.

**TABLE 1:** Family caregivers' characteristics (*N* = 406).

Characteristic	Total (*N* = 406)	Same medical facility as the care recipient		
		Yes (*N* = 186)	No (*N* = 216)	Missing (*N* = 4)
Age (years), *N* (%)				
40–49	13 (3.2)	3 (1.6)	10 (4.6)	0
50–59	114 (28.1)	49 (26.3)	64 (29.6)	1
60–69	198 (48.8)	94 (50.5)	103 (47.7)	1
≥70	81 (20.0)	40 (21.5)	39 (18.1)	2
Gender, *N* (%)				
Men	95 (23.4)	47 (25.3)	46 (21.3)	2
Women	311 (76.6)	139 (74.7)	170 (78.7)	2
Education, *N* (%)				
Did not complete high school	18 (4.4)	7 (3.8)	11 (5.1)	0
High school	189 (46.6)	92 (49.5)	94 (43.7)	3
Career college, junior college, or higher professional school	117 (28.8)	48 (25.8)	69 (32.1)	0
College or graduate school	81 (20.0)	39 (21.0)	41 (19.1)	1
Missing	1			
Annual household income (million JPY), *N* (%)				
<2.50 (about USD 17,000)	145 (35.7)	69 (37.1)	74 (34.4)	2
2.50–4.99	167 (41.1)	78 (41.9)	88 (40.9)	1
5.00–7.99	57 (14.0)	29 (15.6)	27 (12.6)	1
≥8.00	36 (8.9)	10 (5.4)	26 (12.1)	0
Missing	1			
Self‐rated health, *N* (%)				
Poor	28 (6.9)	14 (7.6)	14 (6.5)	0
Not very good	78 (19.2)	35 (19.0)	42 (19.6)	1
Good	242 (59.6)	108 (58.7)	132 (61.7)	2
Very good	54 (13.3)	27 (14.7)	26 (12.1)	1
Missing	4			
Relationship between family caregivers' USC and that of their care recipient				
Same physician	149 (37.1)	149 (80.1)	0	
Same medical facility but different physician	37 (9.2)	37 (19.9)	0	
Different medical facility	216 (53.7)	0	216 (100.0)	
Missing	4			4
JPCAT‐SF, mean (SD)				
Total score	42.1 (16.3)	45.2 (15.6)	40.2 (16.4)	
First contact	27.2 (25.0)	29.6 (25.7)	24.5 (24.5)	
Longitudinality	48.2 (26.2)	53.9 (25.9)	43.9 (25.6)	
Coordination	53.4 (28.5)	54.3 (29.5)	53.5 (27.5)	
Comprehensiveness (services available)	45.9 (27.8)	50.8 (27.4)	42.3 (27.5)	
Comprehensiveness (services provided)	31.1 (30.4)	32.6 (29.9)	31.1 (31.2)	
Community orientation	47.1 (18.0)	50.1 (20.4)	44.9 (20.0)	

Abbreviations: JPCAT‐SF, Japanese version of the Primary Care Assessment Tool Short Form; SD, standard deviation; USC, usual source of care.

### 3.2 Primary care experiences based on the use of the same or a different medical facility

Table 2 summarizes the unadjusted and adjusted associations between family caregivers' use of the same or a different medical facility and their primary care experiences, as assessed by JPCAT‐SF scores. After adjusting for possible confounders, family caregivers who used a different medical facility had lower JPCAT‐SF total scores compared with those who used the same facility (adjusted mean difference −5.73, 95% confidence interval [CI]: −8.93 to −2.54). Among the subscale scores, those for longitudinality, comprehensiveness (services available) and community orientation were significantly lower in the different facility group compared with the same facility group. The largest mean difference was observed for the longitudinality subscale, with an adjusted mean difference of −10.93 (95% CI: −15.98 to −5.89).

**TABLE 2:** Comparison of JPCAT‐SF scores between family caregivers using the same medical facility as their care recipient and those using a different facility (same = 0, different = 1; *N* = 406).

Outcome^a^	Unadjusted mean difference (95% CI)	*p* Value	Adjusted^b^ mean difference (95% CI)	*p* Value
JPCAT‐SF				
Total score	−5.39 (−8.54 to −2.23)	<0.001	−5.73 (−8.93 to −2.54)	<0.001
Subscale scores				
First contact	−4.86 (−9.77 to 0.05)	0.052	−4.79 (−9.68 to 0.10)	0.055
Longitudinality	−10.49 (−15.52 to −5.46)	<0.001	−10.93 (−15.98 to −5.89)	<0.001
Coordination	−1.40 (−7.00 to 4.21)	0.626	−1.88 (−7.55 to 3.80)	0.517
Comprehensiveness (services available)	−8.76 (−14.09 to −3.42)	0.001	−9.41 (−14.85 to −3.98)	<0.001
Comprehensiveness (services provided)	−1.35 (−7.31 to 4.62)	0.658	−1.85 (−7.93 to 4.24)	0.552
Community orientation	−5.51 (−9.47 to −1.54)	0.007	−5.57 (−9.63 to −1.52)	0.007

Abbreviations: CI, confidence interval; JPCAT‐SF, Japanese version of the Primary Care Assessment Tool Short Form.

^a^ Each score was included separately in the model. All scores range from 0 to 100.

^b^ Adjusted for age, gender, educational attainment, annual household income, self‐rated health.

## 4 DISCUSSION

Our study compared the primary care experiences of family caregivers on the basis of whether they used the same medical facility as their care recipient or a different facility. The results revealed that family caregivers who used a different facility from their care recipient reported more negative primary care experiences compared with those who used the same facility, particularly in terms of longitudinality, comprehensiveness (services available), and community orientation. The observed differences in scores for the longitudinality and comprehensiveness (services available) subscales may be explained by the close relationship that is likely to be formed when a family caregiver uses the same medical facility as their care recipient. Using the same facility means primary care providers can recognize them as part of a family unit and provide holistic care. In contrast, when a family caregiver uses a different medical facility from their care recipient, it becomes challenging for providers to recognize their caregiver role, which potentially leads to weaker communication and less comprehensive support. This was consistent with previous studies that reported primary care providers often struggled to identify patients with caring responsibilities, especially when the caregiver and care recipient did not share the same medical facility.^9^, ^10^ In a UK survey, 93% of the surveyed GPs believed GPs should be proactive in identifying caregivers; however, less than half felt confident in doing so.^10^ A survey conducted among family caregivers in Germany found that only 18% had experienced their GP proactively addressing caregiving‐related issues without the caregiver initiating the conversation.^22^ The lack of overlap in the medical facility used may reduce opportunities for primary care providers understand the relationship between a caregiver and their care recipient. In turn, this may contribute to disadvantages in caregivers' primary care experiences, particularly in terms of longitudinality and comprehensiveness (services available).

The lower community orientation scores reported by family caregivers using a different medical facility from their care recipient in this study suggested that integration between primary care providers and broader social and professional support systems may be compromised when family caregivers and their care recipients do not share the same medical facility. When both parties use the same facility, caregivers are more likely to be exposed to community resources and healthcare providers who understand local needs and challenges, which may explain the higher scores we observed for community orientation.

To our knowledge, this is the first study to compare family caregivers' primary care experiences on the basis of whether they used the same medical facility as their care recipient or a different facility. A previous study indicated that family caregivers faced a double disadvantage as they experienced lower health‐related quality of life and poorer primary care experiences compared with noncaregivers.^6^ Therefore, it is important to improve family caregivers' primary care experiences. The results of this study suggested that when family caregivers do not use the same medical facility as their care recipient, their experiences of longitudinality, comprehensiveness (services available), and community orientation are compromised. These results highlighted the need to improve these areas of family caregivers' primary care experiences. Efforts to enhance these experiences could begin with healthcare professionals asking whether patients are responsible for someone's care. Identifying patients with caring responsibilities in clinical practice is most effective when caregiver support is recognized as a priority by the entire healthcare team.^23^

This study had several limitations. First, the cross‐sectional design limited our ability to establish causality between caregivers' primary care experiences and the use of the same or a different medical facility. Longitudinal studies are needed to confirm whether these relationships persist over time. Second, we did not collect detailed information on the characteristics of medical facilities, such as whether they were hospital‐based or community‐based practices.^19^ Further research could explore whether different types of facilities influenced family caregivers' primary care experiences. Third, as this study was conducted in a single prefecture in Japan, caution is warranted regarding its generalizability to other settings.

In addition, this study did not address the reasons why family caregivers used a different medical facility from their care recipient. To our knowledge, there is limited literature on this topic. Further research is therefore needed to clarify these reasons; for example, whether this relates to simple geographic or transportation factors, differences in medical needs, or personal preferences. Understanding these underlying reasons could also help primary care providers tailor support to family caregivers' specific needs.

## 5 CONCLUSIONS

Our study found that family caregivers who used a different medical facility from their care recipient reported more negative primary care experiences than those that used the same facility, particularly in terms of longitudinality, comprehensiveness (services available), and community orientation. These findings indicate that greater efforts are needed to provide patient‐centered, family‐oriented primary care for family caregivers who use a different facility from their care recipient.

## AUTHOR CONTRIBUTIONS

**Gen Nakayama:** Conceptualization (lead); investigation (lead); methodology (lead); formal analysis (lead); writing – original draft (lead); writing – review and editing (lead). **Shoichi Masumoto:** Investigation (supporting); funding acquisition (lead); writing – review and editing (equal). **Junji Haruta:** writing – review and editing (equal). **Tetsuhiro Maeno:** writing – review and editing (supporting).

## CONFLICT OF INTEREST STATEMENT

The authors have stated explicitly that there are no conflicts of interest in connection with this article.

## ETHICS STATEMENT

Ethics approval statement: The survey was approved by the Ethics Committee of the Institute of Medicine, University of Tsukuba (approval no. 1518‐2). This committee grants approval to studies based on the provisions of the Declaration of Helsinki and Japanese ethical guidelines (Ethical Guidelines for Medical and Health Research Involving Human Subjects). All participants were volunteers and checked the box on the questionnaire indicating their intention to participate.

Patient consent statement: None.

Clinical trial registration: None.
